# Governance models to support patient safety when undergoing maximal effort cytoreductive surgery for advanced ovarian/fallopian tube/primary peritoneal cancer – a joint statement of ACPGBI, ASGBI, AUGIS and BGCS

**DOI:** 10.1111/codi.16016

**Published:** 2022-02-01

**Authors:** Charles Maxwell‐Armstrong, Stephen Dobbs, Gill Tierney, Nick Maynard, Nick Wood, Angelika Kaufmann, Kathryn McCarthy, Balamurali Bharathan, Lisa Bird

**Affiliations:** ^1^ ACPGBI Nottingham University Hospitals NHS Trust Nottingham UK; ^2^ BGCS Belfast Health and Social Care Trust Belfast UK; ^3^ ASGBI University Hospitals of Derby and Burton NHS Foundation Trust University of Nottingham Nottingham UK; ^4^ AUGIS Oxford University Hospitals NHS Trust Oxford UK; ^5^ BGCS Lancashire Teaching Hospitals NHS Foundation Trust Preston UK; ^6^ Leeds Teaching Hospitals NHS Foundation Trust Leeds UK; ^7^ North Bristol NHS Trust Bristol UK; ^8^ Nottingham University Hospitals NHS Trust Nottingham UK; ^9^ University of Birmingham Birmingham UK

## BACKGROUND

The standard of care treatment in advanced ovarian/fallopian tube cancer involves a combination of surgery to achieve complete cytoreduction and platinum‐based chemotherapy. The British and European guidelines for the surgical management of advanced ovarian/fallopian tube cancer advise maximal surgical effort cytoreductive surgery. This may require four‐quadrant surgery including multivisceral resection techniques such as peritoneal stripping, diaphragmatic resection, removal of bulky pelvic/para‐aortic lymph nodes, splenectomy, liver and/or liver capsule resection and bowel resection. It is recognized that the delivery of adequate surgery for ovarian cancer frequently requires gynaecological oncologists to work together with colorectal surgeons and with surgeons from other specialities, including upper gastrointestinal (UGI) surgeons.

This statement sets out a framework for joint working for gynaecological oncologists and colorectal and UGI surgeons. The Royal College of Obstetricians and Gynaecologists’ curriculum for subspeciality training in gynaecological oncology is outside the remit of this document. However, we understand that this is under a cycle of review. As key stakeholders the ACPGBI, ASGBI, AUGIS and BGCS are committed to inputting into this process.

## STATEMENTS

1. Colorectal ± UGI surgical input into gynaecological oncology cases must be formally recognized in job plans and appropriately resourced. It is no longer acceptable for colorectal input into gynaecological oncology cases by colorectal surgeons to be performed pro bono.
2. Gynaecological cancer centres will identify at least one, but ideally two colorectal surgeons with a specific interest in this area of joint working. For RCOG‐approved gynaecological oncology training centres one colorectal surgeon will be responsible for the colorectal training of RCOG gynaecological oncology subspeciality trainees. There will be reciprocal arrangements for gastrointestinal surgery trainees to be supported by gynaecological oncologists to achieve the relevant knowledge and competences in gynaecological disease incorporated in the ‘Intermediate & Final Stage Syllabus’ of the Intercollegiate Surgical Curriculum for General Surgery (Intercollegiate Surgical Curriculum Programme).
3. Published evidence confirms the challenge in accurately predicting the need for colorectal surgical input at ovarian debulking surgery, given the poor predictive value of cross‐sectional imaging. Nevertheless, every effort should be made to involve colorectal surgeons ahead of surgery if this is anticipated. This may take the form of a formal multidisciplinary team discussion or may simply involve joint discussion between consultant colleagues. All associations agree that colorectal and UGI surgeons should only be called to theatre for planned procedures without advance notice in exceptional circumstances.
4. The extent of support required by colorectal and other surgeons for the gynaecological oncology surgery team for the provision of maximal effort cytoreductive surgery for advanced ovarian cancer will vary depending upon the skills and experience of the gynaecological oncologists. These arrangements should be agreed with the colorectal and UGI surgery teams and ratified through appropriate clinical governance processes. In some centres, gynaecological oncologists will perform the majority of bowel surgery independently, whereas in others colorectal surgeons will be involved more frequently. In both models, arrangements for postoperative care and management of complications must be explicitly detailed and agreed with both gynaecological oncology and colorectal/UGI surgical departments.
5. Postoperative management of joint gynaecological oncology/colorectal cases will follow documented enhanced recovery after surgery protocols as agreed locally. Both teams will ensure that communication exists with the duty surgical teams. It is accepted that specific individuals will be unlikely to be able to provide 24/7 cover for this group of patients.
6. Postoperative complications arising in elective patients after major gynaecological oncology resections should be managed in a timely fashion and may require liaison with the general surgery on‐call team. Patients with intra‐abdominal complications should be managed in line with the Trust’s emergency laparotomy pathway with regard to resuscitation and cross‐sectional imaging. Preoperative, operative and postoperative management of these cases requiring emergency laparotomy should be performed in line with the recommendations of the National Emergency Laparotomy Audit (NELA). It is planned that these cases will now form part of the NELA database.
7. Patients undergoing surgery by both gynaecological oncology and colorectal/UGI surgeons should be subject to joint regular audit and morbidity and mortality meetings. Patients will be submitted to national registries where appropriate. It is hoped that such registries will expand in the fullness of time to cover large‐bowel‐related issues and patient reported outcome measure data.

## ETHICAL APPROVAL

No ethics committee approval was required for this work.

## Data Availability

All authors contributed to the writing, read and approved the final manuscript.

## DATA AVAILABILITY STATEMENT

Data sharing is not applicable to this article.

